# Massage and Reiki used to reduce stress and anxiety: Randomized Clinical Trial

**DOI:** 10.1590/1518-8345.1614.2834

**Published:** 2016-11-28

**Authors:** Leonice Fumiko Sato Kurebayashi, Ruth Natalia Teresa Turrini, Talita Pavarini Borges de Souza, Raymond Sehiji Takiguchi, Gisele Kuba, Marisa Toshi Nagumo

**Affiliations:** 1 Post-doctoral fellow, Escola de Enfermagem, Universidade de São Paulo , São Paulo, SP, Brazil. Scholarship holder at Coordenação de Aperfeiçoamento de Pessoal de Nível Superior (CAPES), Brazil.; 2 PhD, Full Professor, Escola de Enfermagem, Universidade de São Paulo , São Paulo, SP, Brazil.; 3 Doctoral student, Escola de Enfermagem, Universidade de São Paulo , São Paulo, SP, Brazil.; 4 Master's student, Instituto de Ciências Biológicas, Universidade de São Paulo , São Paulo, SP, Brazil.; 5 Master's student, Escola de Enfermagem, Universidade de São Paulo , São Paulo, SP, Brazil.; 6 Architect.

**Keywords:** Massage, Therapeutic Touch, Complementary Therapies, Anxiety, Stress, Psychological

## Abstract

**Objective:**

to evaluate the effectiveness of massage and reiki in the reduction of stress and anxiety in clients at the Institute for Integrated and Oriental Therapy in Sao Paulo (Brazil).

**Method:**

clinical tests randomly done in parallel with an initial sample of 122 people divided into three groups: Massage + Rest (G1), Massage + Reiki (G2) and a Control group without intervention (G3). The Stress Systems list and the Trace State Anxiety Inventory were used to evaluate the groups at the start and after 8 sessions (1 month), during 2015.

**Results:**

there were statistical differences (p = 0.000) according to the ANOVA (Analysis of Variance) for the stress amongst the groups 2 and 3 (p = 0.014) with a 33% reductions and a Cohen of 0.78. In relation to anxiety-state, there was a reduction in the intervention groups compared with the control group (p < 0.01) with a 21% reduction in group 2 (Cohen of 1.18) and a 16% reduction for group 1 (Cohen of 1.14).

**Conclusion:**

Massage + Reiki produced better results amongst the groups and the conclusion is for further studies to be done with the use of a placebo group to evaluate the impact of the technique separate from other techniques. RBR-42c8wp

## Introduction

Complementary and Alternative Medicine has become an integral part of health care for the population in North America for the treatment of different ailments^1^. In Brazil complementary therapies are known as Complementary and Integrative Practices (PIC) based on Official Government Notice 971 from the Ministry of Health (2006) and it is made up of group of therapies that includes: acupuncture, auriculotherapy, homeopathy, hydrotherapy, herbal medicine, eastern massage, oriental physical exercises like Tai Chi Chuan, Lian Gong, Qi Gong, amongst others^2^. 

Despite the advances in conventional western medicine, the interest for the use of PIC has increased principally in developed countries. It was observed that in 2012 33.2% of adults in North America used some form of complementary health therapy. The therapies that encompass mind and body that are commonly sought after by adults have been yoga, the use of chiropractic and osteopathic practices, meditation and massage therapy^3^. 

The results of a piece of research conducted in 2007 in the United States allowed us to estimate that 18 million people over the age of 18 had used massage therapy in the last 12 months^4^. The massage therapy has been offered with a preference to hospitalized patients to support the management of symptoms of pain, anxiety and tension^1^. Also, what was shown was a reduction in lower back pain, improvements in work related activities and improvements in the quality of life of the nursing teams in the general hospitals in Brazil^5^.

In the classification of complementary therapies proposed by the *National Center for Complementary and Alternative Medicine* (NCCAM), massage belongs to the sub-category of mind-body therapies. In general, the therapies put pressure on, rub and massage the muscles and other soft tissue in the body. The scientific investigations on massage are preliminary and conflicting, but the studies show benefits in relation to the pain and other associated symptoms. A large part of the evidence suggests that the effects are short term and that the people need to continue having sessions to maintain the benefits that are received^6^.

In post-modern society, the high levels of stress have become such a problem for health that it is very common and what can be seen is that the excessive and continuous effect can compromise health having a triggering effect in the development of innumerable illnesses. They can affect the quality of life and the productivity of human beings which has been generating major interests in relation to causes and the methods for their reduction^7^. 

The principal treatment for ailments that cause mental disorders due to stress and anxiety include psychiatric treatment and pharmacotherapy. Amongst the PIC that can contribute to preventing diseases and reducing the levels of stress and anxiety, one can put the spotlight on massage^8^ and Reiki that has been the subject of research as a complementary energy therapy that can help to strengthen the capacity of the body to cure itself. There is a growing interest amongst nurses for the use of Reiki in assisting patients especially in relation to self-help. Research being integrative and revisionary selected investigations using Reiki to reduce stress, to relax, to treat depression, pain and the healing of injuries^9^. 

Although this method of curing is widely used for a variety of psychological and physical symptoms the proof of its efficacy is not over abundant and is conflicting demanding more studies to investigate its affects^10^.

## Objective

To evaluate the effectiveness of massage and reiki in the reduction of stress and anxiety in clients at the Institute for Integrated and Oriental Therapy in Sao Paulo (Brazil). 

## Material and Method

There was a clinically controlled test conducted in a random way that was parallel with three study groups: Massage + Rest Group (G1), Massage + Reiki (G2) and a Control Group without treatment (G3). For the Massage + Rest Group (G1) the Anmá protocol was applied followed by 10 minutes of rest. The Massage + Reiki group (G2) received the massage protocol and the Reiki treatment. The Control group (G3) did not receive an intervention protocol. 

The sessions took place two times per week totaling eight sessions in one month of services. To guarantee the uniformity of the treatment offered the team of 11 people were trained by the Institute for Integrated and Oriental Therapy (ITIO). They were students studying massage and they conducted the collection of data that was monitored by the teachers who had experience in these techniques. 

The population that was studied was made up of volunteers that sought ambulatory care at the ITIO and for military personnel from the IV-Regional Area Command Force in São Paulo (IV-COMAR) located next to ITIO. The participants fulfilled the following inclusion criteria: having a score between 37 to 119 points based on Vasconcelo's Stress Symptoms List (LSS)^11^. The following people were excluded: pregnant women, participants and military personnel from the IV COMAR that had holidays booked for the period of the research or who were on sick leave, those that started to use anxiolytics and antidepressants at the commencement of the research, those that stated they felt discomfort during the massage sessions or who had some sort of tissue injury in the areas that were being massaged. 

In the recruitment phases 141 volunteers on 30 June 2015 were selected, by the team of teachers that were in charge and 13 student collectors from ITIO were used. Of these 141, only 122 were subject to random blocks, from the program called *Research Randomizer Quick Tutorial* ^12^. The data was tabulated by the team on Excel spreadsheets and was subsequently analyzed using the program SPSS 19.0. The minimum number of 30 participants for the group was determined by the analysis of the sample (with the power of 80% and a level of confidence of 95%). 

In addition to the LSS, a questionnaire was used with socio-demographic data and the Trace State Anxiety Inventory. Amongst the tools used for the evaluation of anxiety, the Trace State Anxiety Inventory (IDATE) is considered the gold standard and has been widely used in different studies for the reduction of anxiety^13^. The anxiety-state varies in intensity and can modify itself in time, referring to acute and momentary situations. On the other hand, anxiety-trace relates to the individual difference and are generally more stable. It is characterized by a tendency to react to situations perceived as threatening or provoking anxiety. 

**Figure 1:**
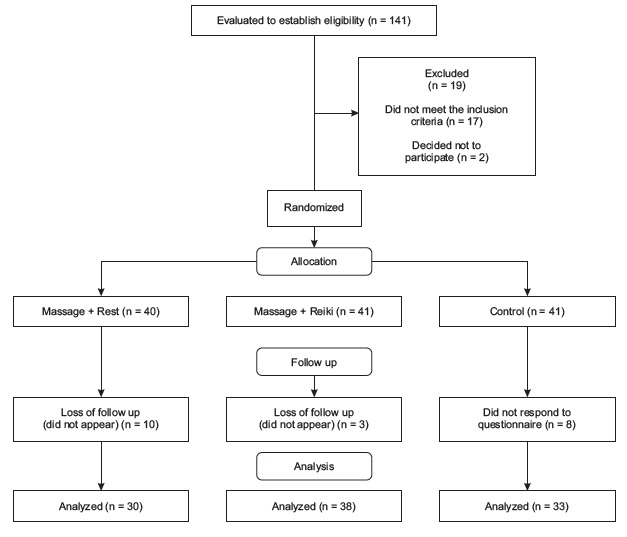
Shows a flowchart of the participants involved in the study in Sao Paulo, Brazil, 2015

Figure 1 shows a flowchart of the participants involved in the study. Twenty-one people left during the course of the study meaning: a loss in the continuity of the treatment (13) and questionnaires that were not responded to (8).

Many of the techniques used in massage and in this study, were focused on the Japanese massage Anmá which is one of the most popular massage techniques in Japan. The Anmá aims to re-balance the energy based on pressure and rubbing in specific areas on the body^14^. 

The use of Anmá involves kneading and putting pressure on the back, neck, thorax, lumbar, gluteus, rubbing the thighs down to the feet (for 20 minutes), using the special meridian line from the bladder and small intestine. The sequence includes: pressure with the palm of the hand on the spine (2 times), pressure on the paravertebral (twice), kneading on the paravertebral (twice), kneading from the start of the spine from the scapula to the top angle of the scapula (twice), pressure on the medium portion of the trapezium (twice), kneading on the medium portion of the trapezium (twice), pressure on the occipital line (twice), kneading with all fingers on cervical spine (3 times), pressure on the gluteus - 2 lines (twice), kneading on the gluteus - 2 lines (twice), pressure on the front part of the thigh - 2 lines (twice), pressure on the popliteal line (twice), kneading in the supra-carpian region on the sural triceps - (twice), pressure on the incept region of the feet - 3 lines (twice) and kneading on the incept region of the feet - 3 lines (twice). Pressure is placed for approximately one second. Kneading is defined as the carrying out of three circular movements when putting on pressure. 

With reference to, Reiki this is a complementary health approach in which the users place their hands lightly on or above the person with the objective of curing the person. Reiki is supposed to mobilize the "vital universal energy" that gives support to the innate and natural abilities of the body and mind supplying force, harmony and equilibrium. Its origins go back thousands of years from Tibet and it was established in 1800 by a Japanese monk called Dr. Mikao Usui^15^. 

The Reiki protocol considered the laying of hands for 2.5 minutes on every position: eyes, occipital region, laryngeal region and on the breastplate on the heart region (during a total of 10 minutes) for the re-equilibrium of the chakras which are important for the mental and emotional state. 

The data was noted down for the relative frequency and the measurements of central tendencies. The comparison between the groups was done through Pearson's chi-squared test and the qualitative variables and the ANOVA to test the average differences. Also, the Levene test was used to check the variance equality. To compare the before and after, the ANOVA test was used for repeated measurements and the Tukey test for multiple comparisons. The size of the effect of the interventions was measured through the Cohen d test and the adopted significance level equaled α = 5%.

The projects theme was "Applicability of the Complementary Practices for the reduction of pain, stress, anxiety and to improve the quality of life" to which the present study is connected which was approved by the Ethics Committee on Research in the School of Nursing at USP (nº 1.105.429/2015). The participants filled in and signed the consent form when being recruited to take part. There were no losses or damages due to the study. 

## Results 

The study finally settled with 101 participants. The majority were female (66%) with a distribution inter-group homogeneity (p = 0.738) and in relation to the activities of the professionals there were military personnel (30), health care professionals (15), students (9) and others (47). The average age of the participants oscillated around 35 years old (Table 1).

**Table 1:** Describes the average and movements from the norm relating to age, stress, anxiety, state, anxiety-trace, according to groups at the initial moment (t_i_), São Paulo, SP, Brazil, 2015

Variable	Group 1	Group 2	Group 3	p*
Age	32.6(11)	35.5(14)	36.7(13.5)	0.450
LSS	66.3(20.6)	67.8(23.4)	69.2(19.7)	0.865
IDATE-state	53.3(7.8)	55.1(10.6)	54.1(10.5)	0.743
IDATE-trace	47.5(7.5)	51.0(11.4)	50.3(10.8)	0.331

* ANOVA (p > 0.05)

The groups showed homogeneity in relation to age and the scores on the LSS and IDATE tools (Table 1).

In Table 2 are the averages and movements away from the norm from the scores from the LSS and IDATE-state before and after treatment. The IDATE-trace was not used in post treatment due to the assumption that the trace does not change with intervention.

**Table 2:** Describes the average and movements from the norm for the stress levels and anxiety-state according to the 3 groups in the initial times (t_i_) and final (t_f_) in São Paulo, SP, Brazil, 2015

Groups	N	LSS - t_i_	LSS - t_f_*	Idate e-t_i_	Idate e - t_f_^†^
G1	30	66.3(20.6)	50.4(21)	53.3(7.8)	44.5(7.8)
G2	38	67.7(23.4)	45.5(22.8)	55.13(10.6)	43.5(9.4)
G3	33	69.2(19.7)	65.9(20.1)	54.09(10.5)	52.30(9.8)
Total	101	67.8(21.2)	53.6(23)	54.3(9.8)	46.7(9.6)

* ANOVA repeated measurements (p < 0.05) ^†^ ANOVA repeated measurements (p < 0.05)

The difference obtained in the LSS-t_f_ were between Groups 1 and 3 (p=0.014) and between Groups 2 and 3 (p = 0.000) as Multiple Comparisons of Tukey. And with reference to anxiety, the differences in the IDATE-E-t_f_ were between Groups 1 and 3 (p = 0.003) and between Groups 2 and 3 (p = 0.000). 

In Table 3 are the sizes of the effect and the percentage of the reduction for each one of the groups and the corresponding classification. 

**Table 3:** Description of the size of the effect (Cohen's d) and the percentage of the change in the evaluation of the LSS and the IDATE and before and after treatment, according to the study groups, São Paulo, SP, Brazil, 2015

LSS	Cohen's d	Percentage	Classification
G1	0.78	-24	Average reduction
G2	0.98	-33	Major reduction
G3	0.17	-5	Small reduction
IDATE-STATE			
G1	1.14	-16	Average reduction
G2	1.18	-21	Average reduction
G3	0.18	-3	Insignificant

Some physical and psychological symptoms of the LSS responded well to the two intervention groups (p < 0.05) through ANOVA for repeated measures and multiple comparisons of Tukey, as per Table 4 as follows. The symptoms that obtained statistical differences for the Intervention Groups (1 and 2) in relation to the Control group (3) were: the feeling that they were about to faint, a lack of energy, no will to do anything, physical exhaustion, headache and an appetite that oscillates. The symptoms that only responded to the Massage and Rest group (1) were: thoughts that caused anxiety, the feeling of wanting to be alone and the feeling of worries.

**Table 4:**
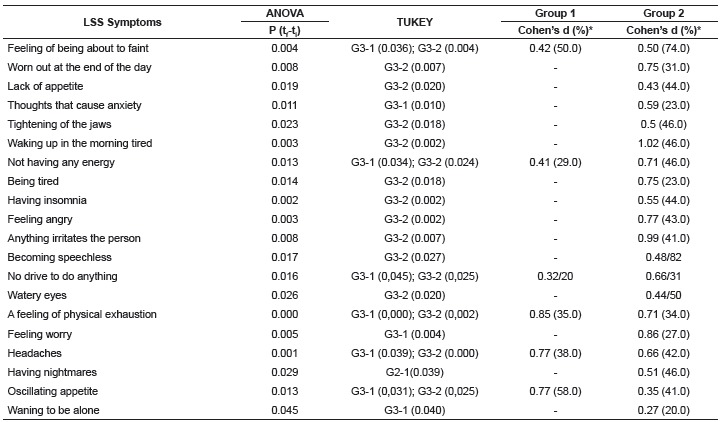
Symptoms from the LSS that obtained significant reductions according to ANOVA of repeated measurements and TUKEY in the 3 groups, Cohen d index and the percentage of reduction in Groups 1 and 2 São Paulo, SP, Brazil, 2015

* The d indexes and the percentage reduction of the symptoms were evaluated before and after the treatment in Groups 1 and 2, after checking which groups had statistical differences (Tukey)

Quantitatively the symptoms that obtained statistical differences and significant reductions were greater for the Massage and Reiki Group (2) in relation to the Control: being worn out at the end of the day, lack of appetite, tightened jaws, waking up tired in the morning, feeling tired, insomnia, anger, irritation, speechless, watery eyes and obscured vison. With reference to the symptoms "having nightmares", only Group 2 was able to improve in relation to Group 1. 

No reports of undesirable effects and injuries were mentioned for each of the groups.

## Discussion

The general stress symptoms were reduced with the treatment demonstrating that stress affects the whole of the body resulting in physiological and psychological changes. With stress the cerebral cortex sends signals to the autonomous nervous system turning the sympathetic into being active. This results in the increase of cardiac frequency and changes in the variability of the frequency reducing the flow of peripheral blood and the flow of renal blood. This increases the blood pressure and the vascular resistance^16^. 

Moderate massaging seems to reduce depression, anxiety, cardiac frequency and the patterns of changes of the electroencephalogram in response to the relaxation that is achieved. This can also lead to an increase in vagal activities and a reduction in the levels of cortisol. In stress data from MRI scans, the scans suggested that the massages using moderate pressure reached the cerebral regions including the amygdala, the hypothalamus, the cingulated cortex and all of the areas involved in stress and emotional regulation^17^.

Massage therapy comprises complementary techniques which can be used as a treatment on its own or associated with other practices which is normally the case in daily clinics being offered to their users. In a study that explored massage therapy combined with meditation it concluded that the results were better for the massage and meditation groups than when compared with the group that received just massage therapy although there were no statistical differences between both groups. The 40 participants in the study were women in the post-operational phase of a mastectomy after having had breast cancer and they showed reductions in the symptoms of stress and anxiety amongst them were: insomnia, a state of alert, fatigue, tension, pain and they referred to relaxation as having given them a better mood and more energy based on the points on the visual analogical scale for each one of the symptoms^18^.

In this study massage associated with reiki increased by 24% the reduction in the stress levels and 16% for the IDATE-state in relation to the Massage and Rest group (G1) also it went to 33.0% for stress and 21.0% for the IDATE-state. 

One can consider that there only being 10 minutes of Reiki post-massage with positive results when it was used in combination (Massage + Reiki) means that an association of these two energy techniques allowed for a reduction to be had in the time for the application of Reiki. The other studies used approximately 20 to 30 minutes in the Reiki sessions as the only technique. In a study where the sessions were 20 minutes the intervention showed positive effects in the reduction of blood pressure being a complementary technique for the control of hypertension^19^. Another study evaluated the effect of 30 minutes of Reiki in the control of anxiety, pain and well-being for patients with cancer (118 patients) in chemotherapy treatment. The evaluations before and after the application of Reiki promoted improvements in well-being, the quality of sleep, relaxation, the alleviation of pain and reductions in the level of anxiety^20^.

The immediate results of Reiki were seen in relation to cardiac beats, the levels of cortisol and the body temperature of professionals with Burnout Syndrome. They suggest that Reiki has an effect on the parasympathetic nervous system when applied to health care professionals^21^. Reiki has an important advantage amongst complementary therapies as it can be self-applied and it was used in this way in research with 20 university students for the program on the reduction of stress and relaxation based on accompanying its effects for 20 weeks^22^. 

Positive symptoms were only seen for the Massage + Reiki Group (G2) relating to physical and emotional state with special attention being drawn to the reduction of nightmares and the reduction of insomnia. However, there is a long journey to be made for the scientific community to recognize the effects of Reiki on its own or as a practice that is integrated with other PIC techniques. A systematic review of Reiki leads us to the conclusion that the evidence is insufficient to say that Reiki is an effective treatment after twenty-three random clinical sessions based on the electronic data^23^. 

Also, the literature is controversial on the issue with reference to the effect of Reiki on the levels of cortisol. In a clinical test with health psychology students no significant changes were found in the levels of cortisol after the application of Reiki^24^. The studies also suggest that one single Reiki sessions is not sufficient to immediately reduce the levels of saliva cortisol. Also, it is not known if there would be changes in the concentrations of saliva cortisol and post-intervention taking place two, six or twenty-four hours on health care professionals with burnout (nurses and doctors)^21^. 

In relation to the limitation of the present study, there was no placebo group for Reiki and it was not possible to compare the effect of the technique in relation to the expectation of the patients. For the next study the placebo could be used where Reiki is not applied. The results provide incentives to carry out new research with evaluations on physiological markers of stress and an evaluation of the time of the Reiki sessions so that the findings can be extended to other populations. 

## Conclusion

Massage and massage combined with Reiki has been shown to be effective in reducing the levels of stress and anxiety. The techniques associated with Massage + Reiki produced better results on the variables of measurements of intervention than when conducted only as Massage + Rest. The symptoms that resulted in positive outcomes in relation to the Massage + Reiki intervention were physical and emotional in nature spotlighting the all-encompassing effects of Reiki. It is therefore suggested that another study with a placebo group for Reiki should be conducted to evaluate the technique on its own. Also, focus should be placed in the use of physiological measures to better evaluate the effects of the techniques on stress and anxiety. 
